# Concomitant histone deacetylase and phosphodiesterase 5 inhibition synergistically prevents the disruption in synaptic plasticity and it reverses cognitive impairment in a mouse model of Alzheimer’s disease

**DOI:** 10.1186/s13148-015-0142-9

**Published:** 2015-10-08

**Authors:** M. Cuadrado-Tejedor, C. Garcia-Barroso, J. Sanzhez-Arias, S. Mederos, O. Rabal, A. Ugarte, R. Franco, M. Pascual-Lucas, V. Segura, G. Perea, J. Oyarzabal, A. Garcia-Osta

**Affiliations:** Neurobiology of Alzheimer’s Disease, Neurosciences Division, Center for Applied Medical Research (CIMA), University of Navarra, Pio XII, 31008 Pamplona, Spain; Small Molecule Discovery Platform, Molecular Therapeutics Program, Center for Applied Medical Research (CIMA), University of Navarra, Pio XII, 55, 31008 Pamplona, Spain; Cajal Institute, CSIC, Madrid, Spain; Department of Biochemistry and Molecular Biology, Faculty of Biology, University of Barcelona, Barcelona, Spain; Bioinformatics Unit, Center for Applied Medical Research (CIMA), University of Navarra, Pamplona, Spain; Anatomy Department, School of Medicine, University of Navarra, Pamplona, Spain

**Keywords:** Histone deacetylase (HDAC), Phosphodiesterase (PDE), Alzheimer’s disease (AD), Memory, Amyloid, Tau, Gene transcription

## Abstract

**Background:**

Given the implication of histone acetylation in memory processes, histone deacetylase inhibitors (HDACIs) have been postulated as potential modulators of cognitive impairment in Alzheimer’s disease (AD). However, dose-dependent side effects have been described in patients with the currently available broad-spectrum HDACIs, explaining why their therapeutic potential has not been realized for chronic diseases. Here, by simultaneously targeting two independent enzyme activities, histone deacetylase (HDAC) and phosphodiesterase-5 (PDE5), we propose a novel mode of inhibitory action that might increase the therapeutic specificity of HDACIs.

**Results:**

The combination of vorinostat, a pan-HDACI, and tadalafil, a PDE5 inhibitor, rescued the long-term potentiation impaired in slices from APP/PS1 mice. When administered *in vivo*, the combination of these drugs alleviated the cognitive deficits in AD mice, as well as the amyloid and tau pathology, and it reversed the reduced dendritic spine density on hippocampal neurons. Significantly, the combination of vorinostat and tadalafil was more effective than each drug alone, both against the symptoms and in terms of disease modification, and importantly, these effects persisted after a 4-week washout period.

**Conclusions:**

The results highlight the pharmacological potential of a combination of molecules that inhibit HDAC and PDE5 as a therapeutic approach for AD treatment.

**Electronic supplementary material:**

The online version of this article (doi:10.1186/s13148-015-0142-9) contains supplementary material, which is available to authorized users.

## Background

There is now considerable evidence that epigenetic regulation is a dynamic and critical mechanism modulating neuronal function. It was recently suggested that memory impairment in Alzheimer’s disease (AD) is caused by a “reversible” epigenetic blockade of gene transcription [1]. One epigenetic mechanism that regulates gene transcription is histone acetylation, a modification that is known to enhance or constrain cognitive functions. In fact, from a therapeutic point of view, histone deacetylase inhibitors (HDACIs) have emerged as promising compounds that might help manage AD. However, the toxicity associated with HDACIs has limited the clinical data available, a phenomenon that may have an important impact in patients receiving long-term therapy [2]. In order to reduce side effects while maintaining cognitive benefits, one strategy would be to develop isoform-selective HDACIs. However, since the side effects reported for HDACIs in humans are dose dependent [3], the use of lower doses of pan-HDAC inhibitors to reduce their toxicity could represent another solution, combining them with other AD-related drugs to obtain a compound or synergistic effect.

Targeting multiple elements in the network underlying AD may produce benefits beyond those of representative monotherapies. Accordingly, for the first time, we propose here a new therapeutic approach to treat AD that targets two independent but synergistic pathways related to different aspects of the disease. Specifically, we propose combining the inhibition of HDACs with that of phosphodiesterase-5 (PDE5), an enzyme that targets another intracellular pathway involved in memory formation and other AD-related features [4–6]. PDE5 inhibitors regulate signalling pathways by elevating cGMP, and they may ultimately promote gene transcription by directly and/or indirectly activating binding to the cAMP response element (CREB) [7]. CREB plays an essential role in regulating the transcription of genes involved in the consolidation of long-term memories [8, 9]. Moreover, its interaction with the CREB-binding protein (CBP) improves memory and enhances synaptic plasticity through HDAC inhibition [10]. Hence, the activation of CREB would be expected to further potentiate the enhancement of memory and synaptic plasticity induced by HDACIs [11]. In line with this idea, PDE5 inhibitors could drive the gene transcription induced by histone acetylation to favour that of CREB-dependent memory-related genes, avoiding global transcriptional activation. Likewise, and due to “epigenetic priming” [12], HDACIs may sensitize the cell’s response to PDE5 inhibitors, facilitating the transcription of CREB-dependent genes and thereby improving therapeutic selectivity.

To assess the potential therapeutic value of concomitant HDAC and PDE5 inhibition in AD, we used the HDACI vorinostat and the PDE5 inhibitor tadalafil as reference compounds. A synergism between these two inhibitors when co-administered at sub-effective doses was not only achieved in terms of memory function but also in reducing the amyloid pathology. Thus, the concomitant inhibition of HDAC and PDE5 may represent a novel symptomatic and disease-modifying strategy to treat AD.

## Results

### The concomitant inhibition of HDAC and PDE5 has a synergistic effect on histone acetylation

**Fig. 1:**
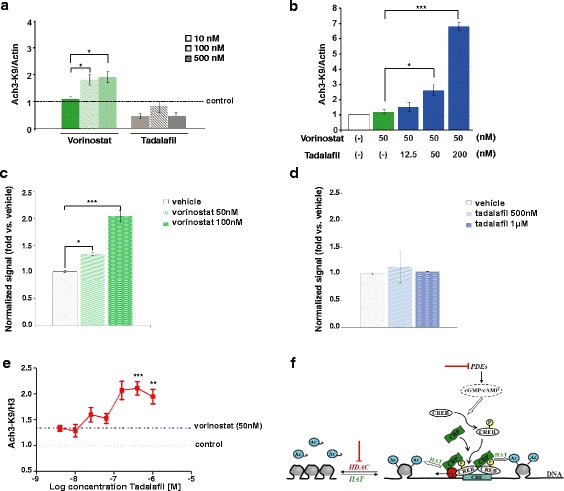
A synergistic effect was observed with the combination of vorinostat and tadalafil on histone 3 acetylation. **a** Western blot analysis showing histone 3 acetylation (AcH3-K9) levels in primary neuronal culture treated with vorinostat or tadalafil for 2 h (*p ≤ 0.05). Actin was used for normalization. **b** Western blot analysis showing AcH3-K9 levels in primary neuronal culture treated with vorinostat and different concentrations of tadalafil for 2 h (*p ≤ 0.05, ***p ≤ 0.001). Actin was used for normalization. **c**–**e** Detection of cellular AcH3-K9 was assayed using SHSY-5Y cells and AlphaLisa technology. SHSY-5Y cells were treated with vorinostat or tadalafil in **c** and **d** or the combination in **e** for 2 h (**p ≤ 0.01, ***p ≤ 0.001). Histone 3 (H3) was used for normalization. In all figures, data are represented as mean ± SEM expressed as fold change versus control vehicle-treated cultures (*n* = 3–4 per condition). **f** Scheme showing a possible synergistic effect with HDAC and PDE inhibition. *Ac* acetyl, *HDAC* histone deacetylase, *HAT* histone acetyl transferase, *CRE* cAMP responsive element, *CREB* CRE-binding protein, *CBP* CREB-binding protein, *P* phospho, *PDE* phosphodiesterase.

Using primary neuronal cultures, we tested whether the combination of vorinostat (a class I and class IIb HDAC inhibitor) and tadalafil (a PDE5 inhibitor) had a synergistic effect on the induction of histone 3 acetylation at lys9 (AcH3K9), an epigenetic mark implicated in memory enhancement in mice [13, 14]. When we exposed the cultured neurons to different concentrations of vorinostat and tadalafil for 2 h (10, 100 and 500 nM), we detected a strong induction of AcH3K9 by vorinostat at concentrations of 100 and 500 nM, whereas no effect was found with tadalafil (Fig. 1a). Interestingly, we observed a synergistic effect on this epigenetic mark when vorinostat (50 nM) was administered in combination with tadalafil (50 and 200 nM: Fig. 1b). This synergistic effect was confirmed in the SH-SY5Y neuroblastoma cell line and using alpha technology, where AcH3K9 was induced significantly by vorinostat (50 nM) combined with tadalafil (from 160 nM: Fig. 1e; compared with each compound alone in Fig. 1c, d). These data suggest that PDE5 inhibition may provoke CREB phosphorylation and, in turn, the recruitment of CBP, thereby enhancing histone acetylation and memory-related gene transcription (Fig. 1f).

### Concomitant inhibition of HDAC and PDE5 had a synergistic effect on long-term potentiation in APP/PS1 transgenic mice

**Fig. 2:**
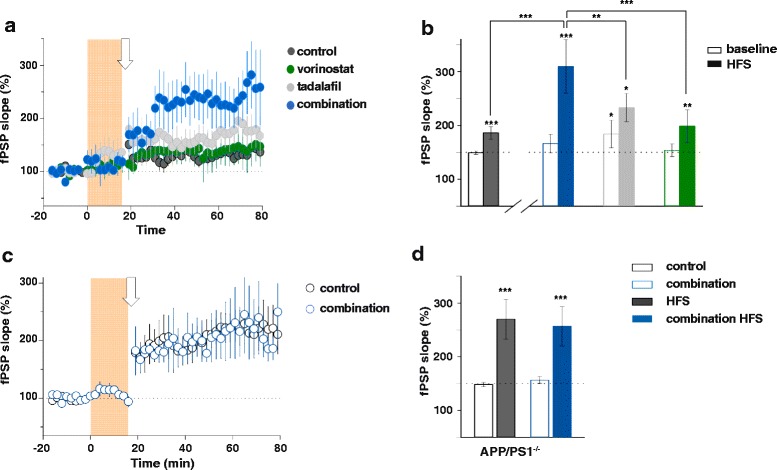
A synergistic effect was observed with the combination of vorinostat and tadalafil on long-term potentiation (LTP) in APP/PS1 mice slices. **a** Relative fPSP slope (from basal values) versus time in hippocampal APP/PS1-positive slices. *Orange vertical bar* denotes time for vorinostat (2 μM ) and/or tadalafil (50 nM) incubation; *arrow* corresponds to the high-frequency stimulation (HFS) protocol. **b** Average relative changes of fPSP slope before and 60 min after HFS in control (*dark grey*; *n* = 6) and vorinostat (*green*; *n* = 5), tadalafil (*light grey*; *n* = 4), and vorinostat and tadalafil (combination, *blue*; *n* = 5) treatments. **c** Relative fPSP slope (from basal values) versus time in APP/PS1-negative slices in control (*black*) and the combination of vorinostat and tadalafil (*blue*) conditions. *Orange vertical bar* denotes time for the combination of vorinostat and tadalafil (combination) incubation; *arrow* corresponds to HFS. **d** Average relative changes of fPSP slope before (*open bars*) and 60 min after HFS (*filled bars*) in control (*black*) and combination treatment (*blue*) in APP/PS1-negative mice (*n* = 8 and 3 slices, respectively); *p ≤ 0.05, **p ≤ 0.01, ***p ≤ 0.001. Data are represented as means ± SEM

To evaluate the physiological consequences of vorinostat and tadalafil treatment, we first investigated their effects by monitoring long-term synaptic plasticity (LTP), a well-known cellular mechanism that underlies memory processes [15]. Extracellular synaptic activity was recorded in hippocampal slices from APP/PS1 adult mice (7–9 months), showing that the LTP induction protocol evoked similar synaptic potentiation after a brief application of vorinostat (2 μM) to that of control slices treated with the vehicle alone. Likewise, tadalafil (50 nM) preincubation did not significantly affect the degree of LTP in APP/PS1 slices (Fig. 2a, b), yet incubation with a combination of vorinostat and tadalafil did produce robust potentiation of synaptic transmission (Fig. 2a, b), significantly beyond that obtained by application of each inhibitor alone. By contrast, the combination of both inhibitors did not affect LTP in APP/PS1-negative slices compared with control conditions (Fig. 2c, d). Therefore, HDAC and PDE5 inhibitors would appear to have a synergistic effect on LTP in AD mice.

### The effects of vorinostat and tadalafil combination therapy on memory function in aged Tg2576 mice

A 2–3-week treatment with vorinostat (50 mg/kg) restored contextual memory deficits in the APPswe/PS1dE9 AD mouse model [16], and likewise, tadalafil (15 mg/kg) improves memory deficits in an aged AD mouse model [17]. Thus, we tested whether the combined administration of lower doses of both of these compounds could restore cognitive deficits in the Tg2576 AD mouse model. To test this hypothesis, we treated 14–16-month-old Tg2576 mice for 4 weeks with tadalafil (1 mg/kg, p.o.) and vorinostat (12.5 mg/kg, i.p.), with either inhibitor alone (tadalafil [1 mg/kg, p.o.] or vorinostat [12.5 mg/kg, i.p.]), or with the vehicle alone (10 % DMSO) daily. We performed an initial pharmacokinetic study of the administration of vorinostat, tadalafil or combined vorinostat and tadalafil at these doses in plasma and brain samples. The brain/plasma ratios 20 min after combined vorinostat and tadalafil administration were 11 % for tadalafil and 5.5 % for vorinostat, corresponding to brain concentrations of 30 and 345.5 nmol/kg (Additional file 1: Figure S1 and Table S1).

**Fig. 3:**
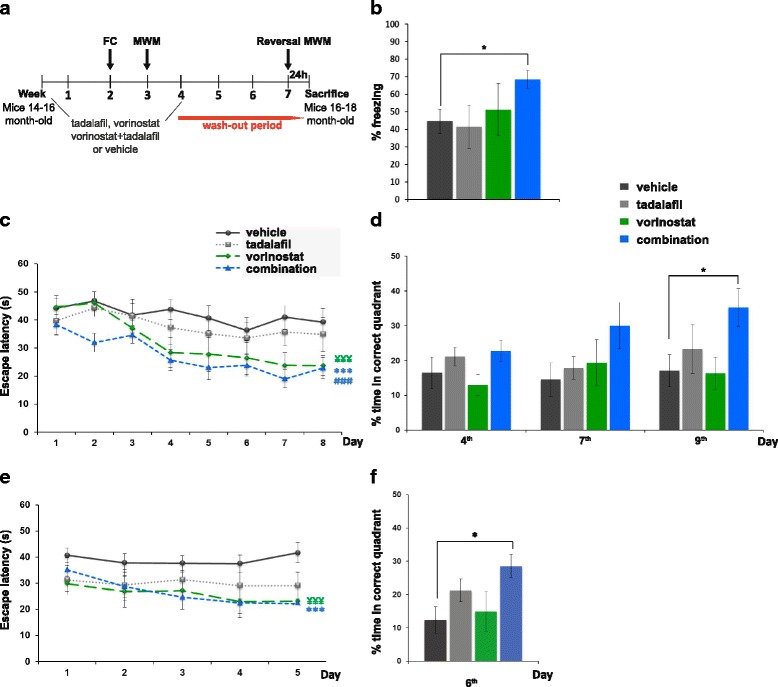
Chronic treatment (4 weeks) with the combination of vorinostat and tadalafil reversed learning deficits in aged Tg2576 mice. **a** Scheme showing timeline for treatment, behavioural tasks and sacrifice of mice. *FC* fear conditioning, *MWM* Morris water maze. **b** Freezing behaviour from Tg2576 mice treated with vehicle, vorinostat, tadalafil or the combination (vorinostat and tadalafil) (*p ≤ 0.05). Data represent the percentage of time freezing during a 2-min test. In this and all subsequent figures, results are expressed as mean ± SEM (*n* = 10–12 per group). **c** Escape latency of the hidden platform in the MWM test for the Tg2576 mice treated with vehicle, vorinostat, tadalafil or combination (***p ≤ 0.001, Tg2576 mice treated with the combination versus Tg2576 vehicle; ^###^ *p* ≤ 0.001, Tg2576 mice treated with the combination versus Tg2576 tadalafil; ^¥¥¥^ *p* ≤ 0.001, Tg2576 treated with vorinostat versus Tg2576 vehicle). **d** Percentage of time spent in correct quadrant of the probe test (days 4, 7 and 9) (*p ≤ 0.05). **e** Escape latency in the reversal MWM test for the Tg2576 mice treated with vehicle, vorinostat, tadalafil or combination after the washout period (***p ≤ 0.001 Tg2576 mice treated with the combination versus Tg2576 vehicle) (^¥¥¥^ *p* ≤ 0.001 Tg2576 treated with vorinostat versus Tg2576 vehicle). **f** Percentage of time spent in correct quadrant during the probe test (day 6) (*p ≤ 0.05)

Memory function was assessed during the second and third week of treatment using two hippocampal-dependent tasks, contextual fear conditioning (FC) and Morris water maze (MWM) tests, respectively (see scheme in Fig. 3a). Compared to wild-type (WT) mice, Tg2576 mice exhibited severe disruption in freezing behaviour in the FC test 24 h after training (*t*(19,2) = 5.3, *p* ≤ 0.001) (Additional file 1: Figure S2a). Memory impairment was rescued to a significant extent by a combined 2-week treatment with vorinostat and tadalafil (*F*(3,44) = 2.7, *p* ≤ 0.05), whereas neither of these drugs alone restored fear memory (Fig. 3b). In the MWM, no significant differences were observed among the experimental groups during the visible-platform training phase, indicating that the animals had a comparable ability to perform the task (Additional file 1: Figure S2b). However, Tg2576 animals (14–16 months old) showed severe cognitive impairment in both the acquisition phase (significant main effect of genotype *F*(1,29) = 13, *p* ≤ 0.001) and during the probe test on days 7 (*t*(29) = 2, *p* = 0.05) and 9 (*t*(29) = 2.2, *p* ≤ 0.05: Additional file 1: Figure S2c and 2d). Notably, the combination of vorinostat and tadalafil rescued these memory deficits, evident through the lower escape latencies in the acquisition phase (*F*(3,316) = 21.3, *p* ≤ 0.001: Fig. 3c). Interestingly, the group receiving only vorinostat also had lower escape latencies compared to the mice that received the vehicle alone (*F*(3,316) = 21.3, *p* ≤ 0.001: Fig. 3c). Nevertheless, during the retention phase on day 9, only the animals that received the combination of vorinostat and tadalafil spent significantly more time than vehicle-treated animals in the correct quadrant (*F*(3,36) = 2.9, *p* ≤ 0.05), demonstrating a synergistic effect of the two drugs on spatial memory retention (Fig. 3d).

Finally, the drug treatments were followed by a washout period of 4 weeks, after which mice were re-trained in a reversal phase of the MWM test, placing the platform in the opposite quadrant. The hidden platform training was carried out over 5 days (four trials per day) and followed by a memory retention probe test on day 6. Tg2576 mice treated with the vehicle performed worse than WT mice in both the acquisition (*F*(1, 28) = 7.0, *p* ≤ 0.01: Additional file 1: Figure S2e) and the retention phase on day 6 (*t*(29) = 2.8, *p* ≤ 0.01: Additional file 1: Figure S2f). By contrast, these transgenic mice treated with the combination therapy displayed a significantly shorter escape latency than the control Tg2576 mice that received the vehicle alone (*F*(3,196) = 10.5, *p* ≤ 0.001: Fig. 3e). Once again, the animals that received vorinostat alone also showed lower escape latencies than the Tg2576 mice that receive the vehicle alone (*F*(3,196) = 10.5, *p* ≤ 0.05: Fig. 3e). During the probe test, only the group of animals receiving the combination of vorinostat and tadalafil spent significantly more time in the target quadrant compared with the vehicle-treated mice (*F*(3,36) = 4.0, *p* ≤ 0.05: Fig. 3f). Together, these results indicate that combination therapy of vorinostat and tadalafil during 4 weeks restored memory impairment in aged Tg2576 mice whose cognition was severely affected. Furthermore, this effect was maintained after a 4-week washout period. Interestingly, the treatment with vorinostat enhances the learning capacity of transgenic mice, yet memory was not consolidated by this drug alone since no differences were detected between Tg2676 mice receiving the vehicle alone or vorinostat during the probe tests.

### Effects of vorinostat and tadalafil combination therapy on pathological AD markers in aged Tg2576 mice

**Fig. 4:**
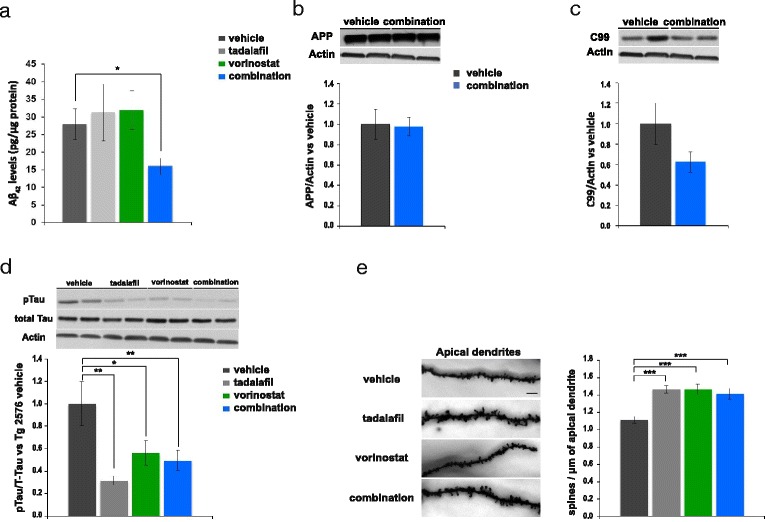
Chronic treatment (4 weeks) with the combination of vorinostat and tadalafil reversed the AD histopathological markers in aged Tg2576 mice. **a** Aβ42 levels determined by ELISA in parieto-temporal cortex of Tg2576 mice treated with vehicle, vorinostat, tadalafil, or combination (vorinostat and tadalafil) (*n* = 8–10 per group) (*p ≤ 0.05). In this and all subsequent figures, results are expressed as mean ± SEM. Effects of chronic administration of vorinostat and tadalafil on full-length APP (**b**) or C-terminal fragment C99 (**c**) levels, normalized to actin, in SDS parieto-cortical extracts. The histogram shows the quantification of the immunochemically reactive bands in the Western blot (representative bands are shown). Data are expressed as the fold change versus Tg2576 mice receiving vehicle (*n* = 8–10 in each group). **d** Representative Western blot bands using the antibody AT8 (pTau), normalized to total tau (T46) of cortical tissues are shown. The histograms represent the quantification of the immunochemically reactive bands in the Western blot. Data are expressed as the fold change versus Tg2576 mice receiving vehicle (*n* = 8–10 in each group) (*p ≤ 0.05; **p ≤ 0.01). **e** Representative Golgi staining images of apical dendrites on CA1 hippocampal pyramidal neurons. *Scale bar*, 10 μm. The histograms represent the quantification of spine density of apical dendrites on hippocampal CA1 pyramidal neurons from Tg2576 mice treated with vehicle, vorinostat, tadalafil or combination (vorinostat and tadalafil) (*n* = 34–36 neurons from three to four animals per group) (***p ≤ 0.001)

Aβ42 was assessed in protein extracts from the parieto-temporal cortex of Tg2576 treated mice by ELISA, and a marked decrease in Aβ42 was observed in the mice that received the combination therapy compared to those that received the vehicle alone (*F*(3,36) = 3.3, *p* ≤ 0.05: Fig. 4a). By contrast, no significant differences were found between the mice that received vorinostat or tadalafil and those treated with the vehicle alone (Fig. 4a). Based on the significant decrease in Aβ42 in the animals that received the combination therapy, we explored APP processing in Western blots of the same extracts. No significant differences were found in APP (Fig. 4b) or in the APP C-terminal C99 fragment (CTF: Fig. 4c) between the Tg2576 mice that received the combination therapy or the vehicle alone. We also analyzed the tau phosphorylation in the same extracts using a pTau-specific (Ser-202/Thr-205) antibody normalized to total tau (T46). Compared to WT mice, there was a significant increase in pTau levels (*t*(7,1) = −3.3, *p* ≤ 0.01: Additional file 1: Figure S3a) in Tg2576 mice, which decreased following vorinostat treatment (*F*(3,11) = 8.7, *p* ≤ 0.05: Fig. 4d). This effect was even more pronounced in the mice that received tadalafil or the combination therapy (*F*(3,11) = 8.7, *p* ≤ 0.01). No significant changes were observed in total tau relative to actin among the experimental groups.

We also tested whether the behavioural recovery induced by the combination therapy reflected structural changes, such as the density of dendritic spines. The density of spines on hippocampal CA1 pyramidal neurons was analyzed in Tg2576 mice and their WT littermates using an optimized Golgi impregnation method. In line with previous findings [18], a significantly lower density of apical dendrites was found on CA1 pyramidal neurons in Tg2576 mice than in WT mice (*t*(70) = 3.9, *p* ≤ 0.001: Additional file 1: Figure S3b), while treatment with tadalafil, vorinostat or the combination of these drugs reversed the deficit in spine density on apical CA1 dendrites, which returned to control values (*F*(3,141) = 12.6, *p* ≤ 0.001: Fig. 4e). These results suggest that the reduction in spine density is reversible even long after disease onset, which might account for the memory improvement observed and maintained for 4 weeks after washout. However, other mechanisms may influence memory recovery since the effect on dendritic spine density in the mice that receive tadalafil alone was not correlated with the behavioural data.

### Gene expression induced by vorinostat, tadalafil and the combination of the two in the hippocampus of Tg2576 mice

To better define the pathways and networks enhanced by the different treatments, we adopted a non-parametric approach implemented in the Gene Set Enrichment Analysis (GSEA) software to determine the genes upregulated by these drugs [19]. GSEA allowed us to determine the pathways and gene signatures enriched among 95 gene sets selected on the basis of memory function and related to AD. GSEA was carried out on the ranked lists reflecting the changes in the microarray studies performed, and the enriched gene sets included those participating in aging and AD-related cellular processes and pathways (reactome amyloids, aging brain, highly calcium permeable postsynaptic nicotinic acetylcholine receptors, among others), providing evidence for an AD-related phenotype. Except for the mice that received vorinostat, several of the gene sets enriched by the other treatments were connected directly with CREB-dependent genes (M15359, combination versus saline, *p* = 0.008 and tadalafil versus saline, *p* = 0.002; M11370, combination versus saline, *p* = 0.010 and tadalafil versus saline, *p* = 0.045; M19129, combination versus saline, *p* = 0.023). Interestingly, the analysis identified enriched pathways and gene signatures associated with synaptic transmission (M2923) through the genes upregulated in the hippocampus of mice administered the combined therapy (vorinostat and tadalafil, *p* = 0.042), but not the individual treatments alone (vorinostat or tadalafil). This suggests that the transcriptional consequences of targeting HDAC and PDE5 together may underlie the recovery of memory function observed in the treated animals.

**Fig. 5:**
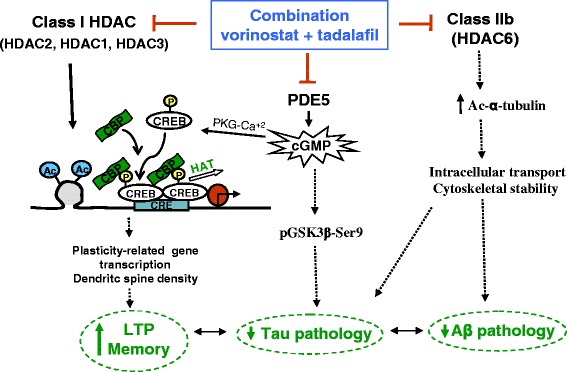
Putative mechanism of action involved in the restoration of pathological signs by the simultaneous inhibition of HDACs and PDE5. *HDAC* histone deacetylase, *Ac* acetyl, *PDE* phosphodiesterase, *PKG* protein kinase G, *LTP* long-term potentiation,  inhibition,  activation

## Discussion

There is convincing evidence that HDACs represent suitable drug targets for therapeutic intervention of AD [16, 20, 21]. However, their adverse side effects mean they are not an option for chronic treatments [22]. While the administration of selective HDACIs represents an attractive potential solution to this challenge [23], counteracting their off-target effects may require sacrificing some of the beneficial effects obtained with current broad-spectrum HDACIs. However, the use of low doses of pan-HDACIs in combination therapy could reduce toxic side effects and might synergistically enhance efficacy. Here, we demonstrate that the co-administration of sub-effective doses of tadalafil, a safe and well-tolerated specific PDE5 inhibitor, together with the HDACI, vorinostat, produces a synergistic effect that prevents the disruption in synaptic plasticity displayed in AD mice. The amelioration in memory impairment exhibited by the treated Tg2576 mice was accompanied by a significant reduction in amyloid and tau pathologies, and the recovery of dendritic spines. Indeed, this combination therapy induces a distinct transcriptional profile in the hippocampus of transgenic mice that may underlie the restoration of the AD phenotype.

Chromatin remodelling due to changes in histone acetylation induces the transcription of genes that encode proteins involved in the growth of new synapses and the increase in synaptic strength [24, 25]. The enhancement of hippocampus-dependent memories by HDACIs required CREB and its interaction with CBP [10]. Indeed, a novel HDACI, crebinostat, enhances memory in mice by facilitating CREB-dependent transcription [11]. Here, we demonstrate a synergistic effect of vorinostat and tadalafil in the induction of the epigenetic response (AcH3K9 levels), suggesting that both the pathways affected by these inhibitors may interact at some point. By elevating cGMP levels, PDE5 inhibition may also produce CREB activation and the recruitment of CBP, a histone acetyltransferase capable of altering chromatin structure [26]. This mechanism could be responsible for restoring LTP and enhancing memory, as observed when the combination of vorinostat and tadalafil was administered to AD transgenic mice. Moreover, the fact that the reversion of memory deficits and synapse loss in aged Tg2576 mice was maintained even after a washout period of 4 weeks suggests that targeting HDAC and PDE5 simultaneously triggers long-lasting changes in plastic remodelling that may be particularly interesting to counteract memory decline in AD.

GSEA data show that the pathways associated with synaptic plasticity are affected by combined treatment with vorinostat and tadalafil and that these effects may be responsible for enhancing memory in Tg2576 mice by augmenting the expression of genes involved in synaptic transmission. The restoration of LTP in APP/PS1 slice mice administered these two inhibitors supports this hypothesis. Interestingly, using a theoretical-biological model that simulates the induction of LTP and of Rubinstein-Taybi syndrome (RTS)-induced LTP deficits, it was predicted that the combination of HDAC and PDE inhibitors would rescue LTP deficits in the RST model [27]. Our results verify the beneficial synergistic effects predicted by this theoretical model.

With regard to AD markers, a reduction in tau pathology was observed in all the mice that received the inhibitors, alone or in combination. Indeed, PDE5 inhibition through the regulation of the Akt/GSK3β pathway is believed to decrease pTau levels [17, 28]. Moreover, the activity of vorinostat on HDAC6 (IC50 10 nM) increases α-tubulin acetylation, possibly one of the mechanisms involved in the amelioration of the tau pathology [29, 30]. Evidence is accumulating that α-tubulin acetylation plays a critical role in the clearance of misfolded and aggregated proteins, since it has been shown to influence aggresome formation as a means of cell protection [31, 32]. Thus, the inhibitory effect of vorinostat on HDAC6 is also likely to participate in the amelioration of amyloid pathology observed with the combination therapy [33, 34]. Nevertheless, a synergistic effect on the transcription of genes involved in amyloid production and/or clearance should also be considered since no effect was observed in the mice that receive vorinostat alone (pan-HDAC inhibitor). Thus, further studies will be necessary to explore the mechanisms involved in the reduction of amyloid and tau pathologies.In summary, the concomitant inhibition of HDAC and PDE5 shown in this study may reverse AD-related pathology through different mechanism of action, some of which remain to be explored (Fig. 5).

## Conclusions

In summary, we propose a new therapeutic approach with potential to treat AD that simultaneously targets HDAC and PDE5. This synergism may make it possible to achieve more optimal safety profiles for HDACIs, making them suitable for chronic treatments. On the one hand, this study suggests that potent HDAC inhibition is not necessary to obtain an efficacious functional response (H3K9 acetylation), and on the other hand, if the changes induced in gene expression underlie the recovery of memory, then simply activating specific gene programmes might be sufficient. Accordingly, molecules with a short half-life and residence time might even produce optimal therapeutic effects. Hence, this may just be the starting point to design and identify molecules with adequate dual activity and that are both efficacious and safe. Moreover, the data presented validate the use of a systems therapeutics approach to drug discovery.

## Methods

### Drugs

For *in vitro* studies, tadalafil (Euroasian Chemicals Private Ltd., Mumbai, India) and vorinostat (Cayman Chemical Company, Ann Arbor, MI, USA) were dissolved in DMSO at 10 mM and to final concentrations in medium cell. For *in vivo* studies, tadalafil (Cialis, Eli Lilly & Company) was administered *in vivo* by oral gavage at a dose of 1 mg/kg and was prepared as previously described [17]. Vorinostat was administered intraperitoneally (i.p.) at a dose of 12.5 mg/kg and was dissolved in 10 % DMSO, 10 % Tween-20 and 90 % saline solution.

### Primary neuronal cultures and treatments

Primary neuronal cultures were obtained from the hippocampus of embryonic day 16 (E16) wild-type (WT) mice and used at 15 days *in vitro* (DIV) [35]. Cultures were treated for 2 h with tadalafil, vorinostat or the combination of vorinostat and tadalafil at different concentrations during 2 h. For Western blot analysis, hippocampal neurons were collected after the different treatments in a cold lysis buffer with protease inhibitors [35].

### Acetyl-Histone H3 Lysine 9 (H3K9ac) cellular detection assay (AlphaLisa technology)

Briefly, 2000 cells (SH-SY5Y) were plated in a poly-d-lysine-treated 384-well plate. Cells were incubated with different concentrations of vorinostat and tadalafil during 2 h. After incubation, the medium was removed and cells were lysed, histones were extracted and histone carrying the acetylation mark was detected following the manufacturer’s instructions (PerkinElmer; Cat number AL714 A/C kit assay). Signal of acetylation mark was obtained after 18 h of dark incubation at room temperature and was normalized by the unmodified histone signal and calculated as folds over basal levels, considered as those obtained in the absence of assayed compounds.

### Slice recordings

Hippocampal slices from APP/PS1 mice, positive and WT littermate mice (6–9 months) were stimulated with bipolar electrodes and recorded with a glass microelectrode placed on the stratum radiatum of the hippocampal CA1 region to monitor extracellular postsynaptic field potentials (fPSPs). Baseline responses were recorded and test stimuli given at 0.1 Hz. Slope of fPSP was analyzed before and after high-frequency stimulation protocol. See the Additional file 1: Additional methods for further details.

### Animals and chronic treatments

Transgenic mice (Tg2576) overexpressing human amyloid precursor protein (hAPP) carrying the Swedish (K670N/M671L) familial AD mutation under control of the prion promoter [36] were used. Mice were on an inbred C57BL/6/SJL genetic background. Animals were housed four to five per cage with free access to food and water, and maintained in a temperature-controlled environment on a 12-h light-dark cycle. Tg2576 female mice (14–16 months old) were treated once daily with tadalafil (1 mg/kg, p.o.), vorinostat (12.5 mg/kg, i.p.), tadalafil (1 mg/kg, p.o.) + vorinostat (12.5 mg/kg, i.p.) (this treatment will be defined as combination therapy), or vehicle for 4 weeks. Behavioural and biochemical studies were performed comparing transgenic mice to age- and strain-matched transgenic negative littermates (WT).

### Behavioural studies

Behavioural studies were carried out during light time (from 9 a.m. to 2 p.m.). Protocols were approved by the Ethical Committee of the University of Navarra (in accordance with the European and Spanish Royal Decree 1201/2005).

#### Fear conditioning test (FC)

To evaluate the effects of drugs on cognitive function after 2 weeks of treatment, fear conditioning paradigm was used as described in [18]. Freezing scores were expressed as percentages. The conditioning procedure was carried out in a StartFear system (Panlab S.L., Barcelona, Spain).

#### Morris water maze test (MWM)

After 3 weeks of treatment, we used the MWM test to evaluate the working and reference memory function in Tg2576 mice, as previously described [17]. In addition, after a 4-week washout period of the drugs, a reversal phase of MWM was carried out. In this phase, the platform was placed in the opposite quadrant of the tank, and the hidden platform training during five consecutive days (four trials per day) was performed. All cues remained in their original positions. Memory retention was analyzed in a probe at day 6. All experimental procedures were performed blind to groups. Animals were euthanized 24 h after the last probe. One hippocampus of three animals per group (vehicle, vorinostat, tadalafil and the combination) was used for RNA extraction, Affymetrix microarray hybridization and data analysis (see Additional file 1: Additional methods).

### Determination of Aβ levels

Parieto-temporal cortical Aβ42 levels were measured by using a sensitive sandwich ELISA kit (Invitrogen, Camarillo, CA). We measured Aβ42 pool containing intracellular and membrane-associated Aβ42 that may be more closely related to the expression of AD signs than other measured Aβ42 species [37]. The tissue was homogenized in a buffer containing SDS 2 %, Tris-HCl (10 mM, pH 7.4), protease inhibitors (Complete Protease Inhibitor Cocktail, Roche) and phosphatase inhibitors (0.1 mM Na3VO4, 1 mM NaF). The homogenates were sonicated for 2 min and centrifuged at 100,000×*g* for 1 h. Aliquots of supernatant were directly diluted and loaded onto ELISA plates in duplicate. The assays were performed according to the manufacturer’s instructions.

### Immunoblotting

For Western blot analysis of APP-derived fragments, SDS 2 % protein extracts were separated in a CriterionTM Tris-Tricine 10–20 % gradient precast gel (Bio-Rad, Hercules, CA, USA). For analysis of pTau and Tau, proteins were separated in a Criterion TM Bis-Tris 4–12 % gradient precast gel (Bio-Rad, Hercules, CA, USA). For further details, see Additional file 1: Additional methods.

### Dendritic spine measurements

A modified Golgi-Cox method was used [18, 38]. For each mouse (*n* = 4 per group), nine neurons (three dendritic segments were measured per neuron, at least 30 μm long) were analyzed.

### Data analysis and statistical procedures

The data was analyzed with SPSS for Windows, version 15.0 (SPSS, Chicago, IL, USA), and unless otherwise indicated, the data is expressed as means ± standard error of the mean (S.E.M.). Normal distribution of data was checked by the Shapiro-Wilk test.

In the MWM, latencies to find the platform were examined by a two-way repeated measures ANOVA test (genotype × trial) to compare the cognitive status in WT mice and Tg2576 mice. Likewise, the treatment effect in spatial memory was examined also by a two-way repeated measures ANOVA test (treatment × trial) followed by post hoc Scheffe’s analysis. In those cases where no interaction was found between factors, the *F* value associated to genotype or treatment main effect will be shown. When two groups were compared, Student’s *t* test was used, whereas when more than two experimental groups were compared, one-way ANOVA followed by post hoc Scheffe’s test was used. Each biochemical assay was repeated, at least, three times, and the data were analyzed using one-way ANOVA followed by post hoc Scheffe’s test.

## Additional file

Additional file 1:
**Additional methods.** **Table S1.** Pharmacokinetic parameters and blood-brain barrier permeability for vorinostat and tadalafil. **Figure S1.** Mean plasmatic concentration evolution versus time after different administrations of vorinostat (SAHA, 12.5 mg/kg, i.p. administration) (**a**), tadalafil (1 mg/kg, oral administration) (**b**) and concomitant vorinostat and tadalafil administration (12.5 mg/kg, i.p. administration, and 1 mg/kg, oral administration, respectively) (**c**). **Figure S2.** Memory tests after chronic treatment with vehicle in non-transgenic mice (WT) compared to transgenic mice (Tg2576). **Figure S3.** (**a**) Representative Western blot bands using the AT8 (pTau) antibody normalized to total tau (T46) in cortical tissues of WT and Tg2576 animals treated with vehicle. The histograms represent the quantification of the immunochemically reactive bands in the Western blot. An increase in pTau levels in Tg2576 mice receiving vehicle are shown compared to WT-vehicle mice. Results are expressed as mean ± SEM (*n* = 8–10 in each group) (***p* ≤ 0.01). (**b**) Representative Golgi staining images of the apical dendrites on CA1 hippocampal pyramidal neurons in WT and Tg2576 animals treated with vehicle. Scale bar: 10 μm. WT vehicle group showed a significantly higher level in the spine density of apical dendrites on hippocampal CA1 pyramidal neurons than Tg2576 vehicle mice (*n* = 34–36 neurons; ****p* ≤ 0.001). (PDF 270 kb)

## Abbreviations

AD: Alzheimer’s disease
APP: amyloid precursor protein
Aβ: amyloid-β
FC: fear conditioning
HDAC: histone deacetylase
LTP: long-term potentiation
MWM: Morris water maze
PDE: phosphodiesterase
