# Supplementary material for: Concomitant histone deacetylase and phosphodiesterase 5 inhibition synergistically prevents the disruption in synaptic plasticity and it reverses cognitive impairment in a mouse model of Alzheimer’s disease
Source: Clin Epigenetics. 2015 Oct 8;7:108. doi: 10.1186/s13148-015-0142-9 (PMC4599811; doi:10.1186/s13148-015-0142-9)
Supplement: Additional file 1: — Additional methods. Table S1. Pharmacokinetic parameters and blood-brain barrier permeability for vorinostat and tadalafil. Figure S1. Mean plasmatic concentration evolution versus time after different administrations of vorinostat (SAHA, 12.5 mg/kg, i.p. administration) (a), tadalafil (1 mg/kg, oral administration) (b) and concomitant vorinostat and tadalafil administration (12.5 mg/kg, i.p. administration, and 1 mg/kg, oral administration, respectively) (c). Figure S2. Memory tests after chronic treatment with vehicle in non-transgenic mice (WT) compared to transgenic mice (Tg2576). Figure S3. (a) Representative Western blot bands using the AT8 (pTau) antibody normalized to total tau (T46) in cortical tissues of WT and Tg2576 animals treated with vehicle. The histograms represent the quantification of the immunochemically reactive bands in the Western blot. An increase in pTau levels in Tg2576 mice receiving vehicle are shown compared to WT-vehicle mice. Results are expressed as mean ± SEM (n = 8–10 in each group) (**p ≤ 0.01). (b) Representative Golgi staining images of the apical dendrites on CA1 hippocampal pyramidal neurons in WT and Tg2576 animals treated with vehicle. Scale bar: 10 μm. WT vehicle group showed a significantly higher level in the spine density of apical dendrites on hippocampal CA1 pyramidal neurons than Tg2576 vehicle mice (n = 34–36 neurons; ***p ≤ 0.001). (PDF 270 kb) [file 13148_2015_142_MOESM1_ESM.pdf]

Additional methods

Slice electrophysiology

Hippocampal slices were obtained from APP/PS1 mice, positive and WT littermates mice (6-9 months). Animals were anaesthetized and decapitated. The brain was rapidly removed, placed in ice-cold modified cutting extracellular solution and sectioned in slices (400 μm). Slices were incubated during >1 h at room temperature (22-24º C) in standard extracellular solution containing (in mM): NaCl 124, KCl 2.69, KH_2PO_4 1.25, $MgSO_4$ 2, $NaHCO_3$ 26, $CaCl_2$ 2, and glucose 10, and was gassed with 95% O_2 /5% CO_2 (pH = 7.35). Cutting solution containing (in mM): N-Methyl-D-glucamin 93, KCl 2.5, NaH_2PO_4 1.25, $NaHCO_3$ 30, Hepes acid 20, Glucose 25, thiourea 2, $C_6H_7NaO_6$ 5, Na-pyruvate 3, $CaCl_2$ 0.5 and $MgCl_2$ 10 (pH = 7.35). Slices were then transferred to an immersion recording chamber and perfused with gassed solution (30-34 ºC) at a rate of 1-2 ml/min. The recording area was visualized under an Olympus BX50WI microscope (Olympus Optical, Tokyo, Japan). Bipolar platinum/iridium stimulation electrode of 50 μm and the recording electrode were placed on *stratum radiatum* of hippocampal CA1 region to record synaptic activity. Recording glass microelectrodes of 3-5 MΩ were filled with NaCl 3 mM. Extracellular postsynaptic field potentials (fPSPs) were amplified (EX1; DAGAN Corporation; Minneapolis, MN), bandpass filtered between 0.3 Hz and 1.0 kHz, and digitized at 10.0 kHz (pCLAMP 9 software; Molecular Devices, LLC. Sunnyvale, CA). A stimulus intensity, which evoked half-maximum amplitude fPSPs, was used. Baseline responses were recorded for at least 10 min with test stimuli given at 0.1 Hz. The initial phase of the fPSP was used to quantify synaptic transmission; the slopes during the experiments are expressed as percentages of the baseline slope. Vorinostat (2 μM), tadalafil (50 nM), or the combination of the aforementioned added to the external solution were perfused 15-20 min before LTP-induction protocol was applied, i.e., high frequency stimulation (HFS) consisting in 100 Hz trains delivered 4 times at 0.05 Hz.

Data are presented as mean +/- S.E.M. Paired *t*-test and one-way ANOVA test were used to detect any significant changes in synaptic activity before and after the HFS and between different groups.

Pharmacokinetic Studies

Pharmacokinetic study of vorinostat (SAHA), tadalafil and concomitant vorinostat and tadalafil administration in plasma samples

Vorinostat and tadalafil were measured in plasma samples using a Xevo-TQ MS triple quadropole mass spectrometer with an electrospray ionization (ESI) source and an Acquity UPLC (Waters, Manchester, UK).

Vorinostat solutions were prepared by dissolving the solid in DMSO and this solution was made up to a final volume by addition of 0.9% NaCl (1/9, v:v:v, DMSO/saline). A vorinostat dosage of 12.5 mg/Kg was administered as a single intraperitoneal injection. Tadalafil solution was administered as Cialis and prepared in the same way, with a dosage of 1 mg/Kg administered orally. For the concomitant administration, vorinostat and tadalafil were administered in the same way (IP and orally respectively). Blood was collected at predetermined times over 24 h post injection (0.17, 0.25, 0.5, 1, 2, 4, 8 and 24 h) into tubes containing citrate and plasma was obtained via centrifugation (4˚ C, 13200 rpm, 5 min) and stored at -80˚ C until analysis.

Chromatographic separation was performed by gradient elution at 0.45 mL/min using a XSelect CSH C18 column (50 x 2.1 mm, 2.5 um particle size; Waters). The mobile phase consisted of A: water with 0.1% formic acid, B: methanol with 0.08% formic acid. The autosampler temperature was set at 10˚ C and column temperature at 40˚ C. For detection and quantification, the electrospray ionization operated in the positive mode, and the collision gas used was ultra-pure argon at a flow rate of 0.15 mL min^{-1}. The analytes and internal standards were detected using multiple reaction monitoring (MRM).

Quantification was achieved by external calibration using matrix-matched standards. Concentrations were calculated using a weighted least-squares linear regression (W = 1/x). Calibration standards were prepared by adding the appropriate volume of diluted solutions of the compound (made in a mixture of methanol and water, 50:50, v:v) to aliquots of 25 µL of blank plasma. 2% formic acid in acetonitrile was added to precipitate the proteins. This protein precipitation solvent contained 10 nM of vorinostat-d5 and tadalafil-d3, the internal standards. The mixture was then agitated for 5 min and centrifuged at 13200 rpm for 10 min at 4˚ C. The resulting supernatants were transferred to a Ostro plate (Waters, Manchester, UK), designed to remove

phospholipids. The resulting eluents were evaporated at 37° C under a stream of nitrogen. Residues were dissolved in 50 µL of a mixture of A and B phases (50:50, v:v). A 1.5 µL aliquot of the resulting solution was injected onto the LC-MS/MS system for analysis. Frozen plasma samples were thawed at room temperature, vortexed thoroughly and subjected to the above described extraction procedure.

The pharmacokinetic parameters were obtained by fitting the blood concentration-time data to a non-compartmental model with the WinNonlin software (Pharsight, Mountain View, CA). (Supplementary Figure 2 and Supplementary Table 1).

Determination of brain to plasma concentration ratios of vorinostat and tadalafil after administration of vorinostat, tadalafil or the combination

Vorinostat was injected (12.5 mg/Kg, i.p.) to mice and 0.25 h after injection plasma and brain samples were collected. A second study was conducted: vorinostat was administered in the same way (12.5 mg/Kg, i.p) and tadalafil was administered orally as Cialis with a dosage of 1 mg/Kg; 0.33 h after injection plasma and brain samples were collected. Samples were frozen at 80° C until further process for analysis.

Chromatographic separation was performed by gradient elution at 0.4 mL/min using an Acquity UPLC BEH C18 column (100 x 2.1 mm, 1.7 µm particle size; Waters). The mobile phase consisted of A: water with 0.1% formic acid, B: methanol. The autosampler temperature was set at 10° C and column temperature at 45° C. Compound detection in plasma and brain samples was carried out as described previously for plasma samples for PK. Quantification and sample preparation in the case of brain samples was similar to that applied to plasma samples. Brain samples were thawed unassisted at room temperature and homogenized using a Branson 250 ultrasonic sonifier (Branson, Danbury, Connecticut, USA). When homogenized, 75 mg were weighted and extracted as described previously, with the difference that residues were dissolved in 100 µL of a mixture of methanol and water with 0.1% formic acid (75:25, v:v).

BBB permeability was reported as logBB, where BB is the ratio of the brain to serum concentration. When only vorinostat was administered, concentration values led to a logBB of -1.17 which indicates that about 6.7% of the compound in the bloodstream

crosses the BBB to reach the brain. According to previous studies performed in our laboratory, when only tadalafil was administered, concentration values led to a logBB of -0.89 (Garcia-Barroso et al. Neuropharmacology, 2013, 64, 114-123). In the case of concomitant vorinostat and tadalafil administration, concentration values obtained determined 20 minutes after administration led to a logBB value equal to -1.3 for vorinostat and -1.0 for tadalafil, which indicate that about 5.5% of vorinostat (SAHA) and 11.0% of tadalafil in plasma cross the BBB; thus, corresponding brain concentrations are 345.5 and 30 nmol/Kg respectively. (Supplementary Table 1).

Immunoblotting

In all cases, the membranes were blocked and incubated overnight with the following primary antibodies: mouse monoclonal 6E10 (amino acids 1–16 of Aβ peptide, 1:1000, Covance, San Diego, CA, USA), mouse monoclonal anti-p-Tau AT8 (1:1000; Thermo Fisher Scientific, Rockford, USA), mouse monoclonal anti-tau (1:5000, clone Tau46, Sigma-Aldrich, St. Luis, MO, USA), rabbit monoclonal anti-acetylated H3-Lys9 (1:1000 Cell Signalling Technology, Beverly, MA) mouse monoclonal anti-actin, (1:20 000, Sigma-Aldrich, St. Louis, MO, USA) in the corresponding buffer. Immunolabelled protein bands were detected by using HRP-conjugated anti-rabbit or anti-mouse antibody (1:5000, Santa Cruz Biotechnology, Santa Cruz, CA, USA) or anti-goat (1:1500, Dako) antibody following an enhanced chemiluminescence system (ECL, GE Healthcare Bioscience, Buckinghamshire, UK).

Affymetrix microarray hybridization and data analysis

The hippocampi were dissected and RNA was extracted with TRIzol Reagent (Invitrogen) according to the manufacturer's instructions. As a last step of the extraction procedure, the RNA was purified with the RNeasy Mini-kit (Qiagen, Hilden, Germany). Before cDNA synthesis, RNA integrity from each sample was confirmed on Agilent RNA Nano LabChips (Agilent Technologies). The sense cDNA was prepared from 300 ng of total RNA using the Ambion® WT Expression Kit. The sense strand cDNA was then fragmented and biotinylated with the Affymetrix GeneChip® WT Terminal Labeling Kit (PN 900671). Labeled sense cDNA was hybridized to the Affymetrix Mouse Gene 2.0 ST microarray according to the manufacturer protocols and using

GeneChip® Hybridization, Wash and Stain Kit. Genechips were scanned with the Affymetrix GeneChip® Scanner 3000. Microarray data files were submitted to the GEO (Gene Expression Omnibus) database and are available under accession number GSE62240.

Both background correction and normalization were done using RMA (Robust Multichip Average) algorithm {Irizarry, 2003 #162}. Then, a filtering process was performed to eliminate low expression probe sets. Microarray batches was hybridized and analyzed applying the criterion of an expression value greater than 16 in at least two samples for each experimental condition (hippocampi from mice treated with vorinostat, tadalafil, vorinostat and tadalafil and vehicle, n=3), 29590 probe sets were selected for statistical analysis. Probe sets corresponding to non-coding genes were filtered. R/Bioconductor {Gentleman #163} was used for preprocessing and statistical analysis. LIMMA (Linear Models for Microarray Data) {Smyth, 2004 #164} was used to calculate the fold-change for each treatment compared with the vehicle group.

Functional and pathway analysis

Enrichment analysis of gene sets was carried out using the fold change ranked list of genes as input to the non-parametric Kolmogorov-Smirnoff rank test as implemented in the GSEA (Gene Set Enrichment Analysis) software {Subramanian, 2005 #161}. Gene categories were selected from MsigDB database performing a query using keywords of interest (learning, creb, amyloid, aging, alzheimer, epigenetic, synaptic and neuron). A total of 95 gene sets were selected for further analyses. The p-values for each gene-set were computed based on 2500 permutation iterations. For each treatment we select those categories enriched with p-value<0.05 in the up-regulated genes.

Additional Table 1. Pharmacokinetic parameters and blood-brain barrier permeability for vorinostat and tadalafil. Pharmacokinetic parameters were estimated by fitting the experimental data to a non-compartmental model using Winnonlin software for pharmacokinetic analysis for vorinostat (12.5 mg/Kg, i.p. administration), tadalafil (1 mg/Kg, oral administration) and concomitant vorinostat and tadalafil administration (12.5 mg/Kg, i.p. administration; and 1 mg/Kg, oral administration, respectively). The parameters are: Area Under the Curve (AUC); half life of the product (T ½); clearance (Cl); volume of distribution (Vz); time to reach maximum plasma concentration (T_{max}); blood-brain barrier permeability (LogBB); brain to plasma concentration ratio (BP ratio).

Parameter	Value			
	Vorinostat (12.5mg/Kg, i.p.)	Tadalafil (1mg/Kg, oral)	Vorinostat&Tadalafil (12.5mg/Kg, i.p.; 1mg/Kg, oral)	
			Vorinostat	Tadalafil
**AUC_{0-inf} (h*nM)**	3361	3084	2502	3190
**T 1/2 (h)[a]**	14.5	7.9	9.5	4.9
**Cl/F (L/h)[b]**	0.42	0.02	0.46	0.02
**Vz/F (L)[b]**	8.8	0.26	6.3	0.14
**Norm. Vz/F (L/Kg)**	295.3	9.5	259.1	5.7
**T_{max} (h)**	0.25	2	0.17	2
**Brain concentration (nmol/Kg)**	396[c]	999[d]	345.5[e]	30.0[e]
**Plasma concentration (nM)**	5894[c]	7788[d]	6444	288e
**LogBB**	-1.17[c]	-0.89[d]	-1.4[e]	-1.0[e]
**BP ratio (%)**	6.7[c]	12.8[d]	5.5[e]	11.0[e]

[a] Half life is terminal half life (Lambda z).
[b]For extravascular models (IP), the fraction of dose absorbed cannot be estimated and therefore, Volume and Clearance for these models are Volume/F and Clearance/F, where F is the fraction absorbed. Volume of distribution is the volume of distribution based on the terminal phase for these models (Vz).
[c] Determined at T_{max}.
[d] Reference: Garcia-Barroso et al. Neuropharmacology, 2013, 64, 114-123 (for a dose of 15 mg/Kg of tadalafil at 90 min)
[e] Determined at 20 min.

Additional Figure S1:

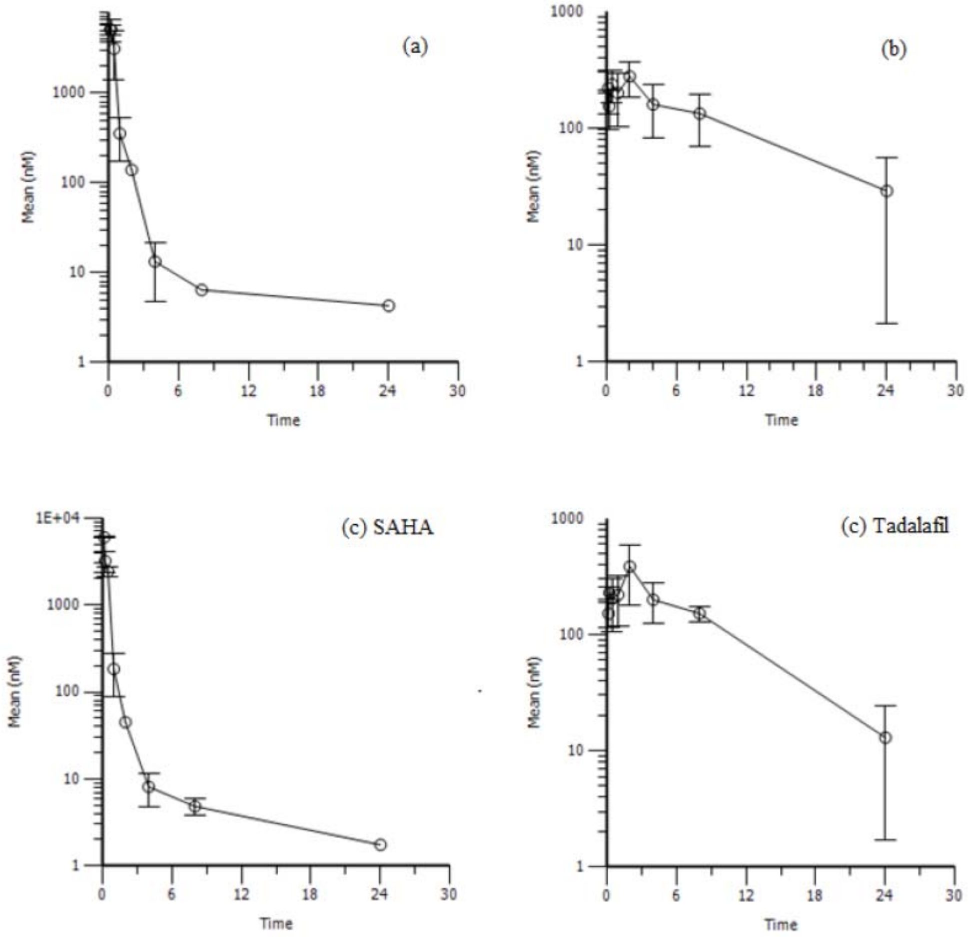

Legend Additional Figure S1: Mean plasmatic concentration evolution versus time after different administrations of vorinostat (SAHA, 12.5 mg/Kg, i.p. administration) (**a**), tadalafil (1 mg/Kg, oral administration) (**b**) and concomitant vorinostat and tadalafil administration (12.5 mg/Kg, i.p. administration; and 1 mg/Kg, oral administration, respectively) (**c**).

Additional Figure S2

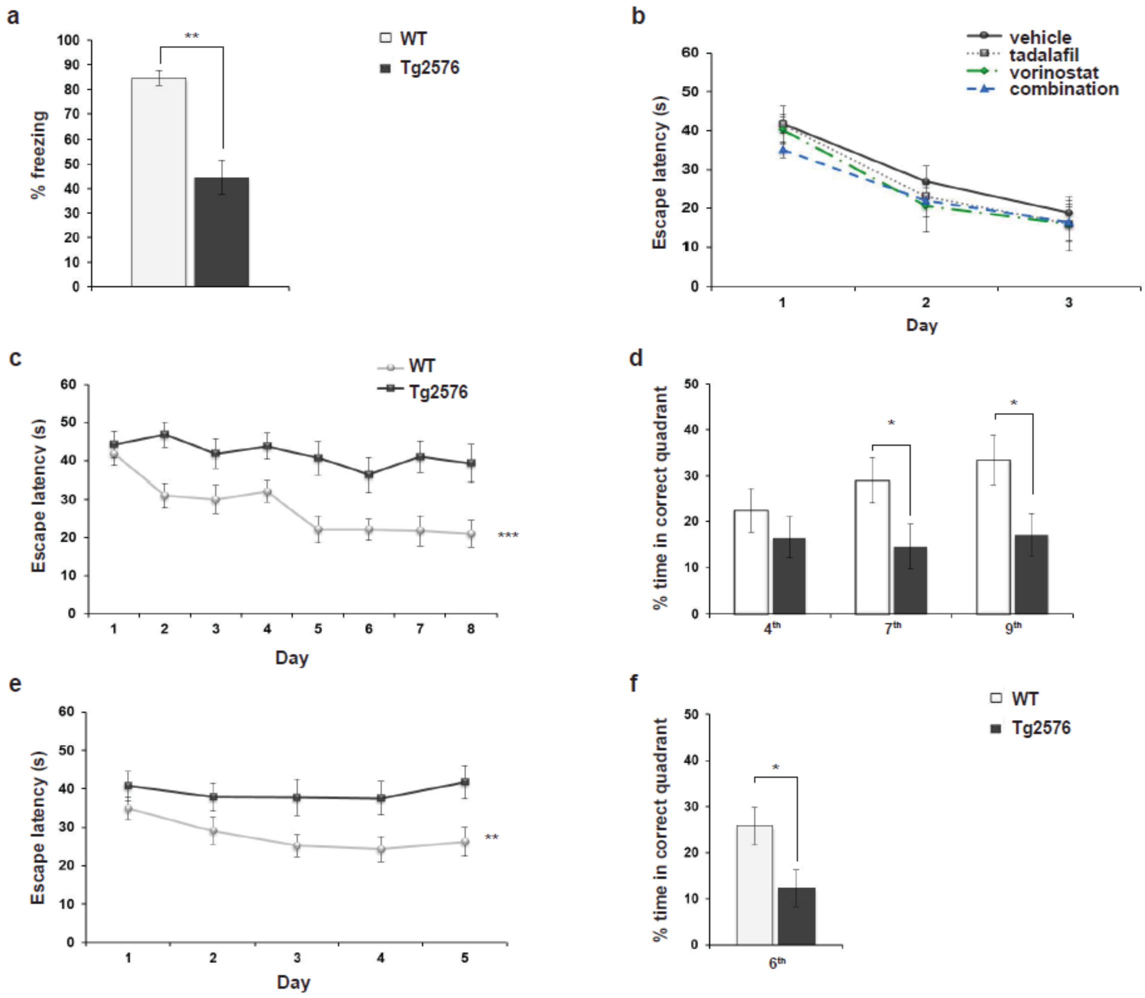

Legend Additional Figure S2. Memory tests after chronic treatment with vehicle in non-transgenic mice (WT) compared to transgenic mice (Tg2576). (**a**). Vehicle-treated Tg2576 mice exhibited significantly less freezing behaviour than WT vehicle counterparts (*** $p \leq 0.001$) in the fear conditioning task. Data represent the percentage of freezing time during a 2 min test. In this and all subsequent figures, results are expressed as mean ± SEM (n=10-12 per group). (**b**) No significant differences in escape latency were detected among groups (Tg2576 treated with vehicle, vorinostat, tadalafil and combination therapy) during any of the 3 days of visible-platform training. (**c**) Escape latency of the hidden-platform in the MWM test for the Tg2576 mice treated with vehicle and WT vehicle mice (*** $p \leq 0.001$). (**d**) Percentage of time spent in correct quadrant during probe test (days 4, 7 and 9). Tg2576 vehicle mice performed significantly worse than WT vehicle mice on days 7 and 9 (* $p \leq 0.05$). (**e**) Escape latency of the reversal MWM test for the Tg2576 mice treated with vehicle versus WT vehicle mice after washout period (** $p \leq 0.01$) (**f**) Percentage of time spent in correct quadrant during the probe on day 6. Tg2576 mice receiving vehicle performed significantly worse than WT mice (* $p \leq 0.05$).

Additional Figure S3

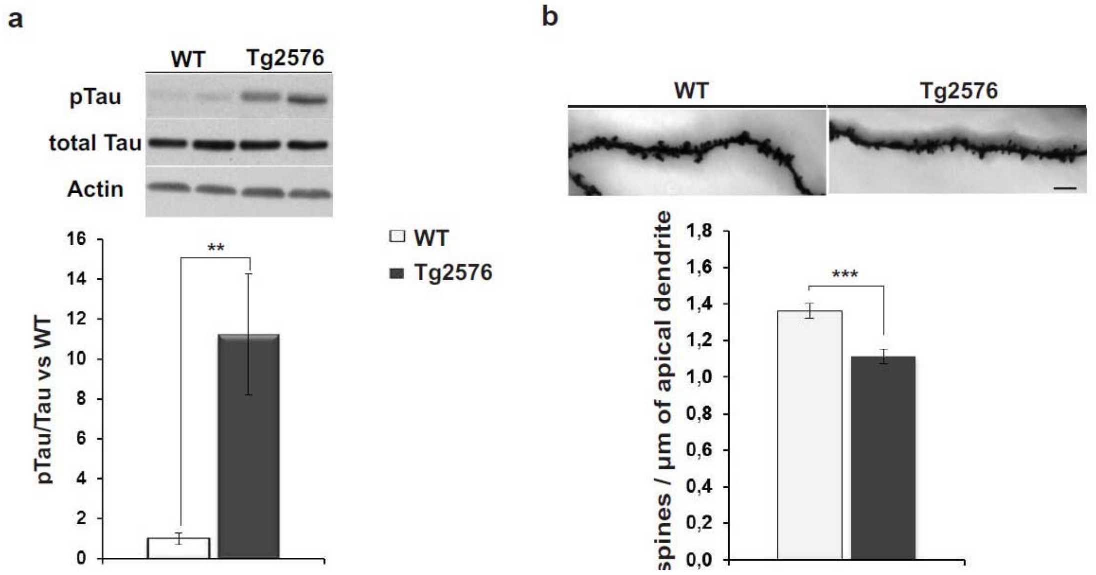

Legend Additional Figure S3. (**a**) Representative western blot bands using the AT8 (pTau) antibody normalized to total tau (T46) in cortical tissues of WT and Tg2576 animals treated with vehicle. The histograms represent the quantification of the immunochemically reactive bands in the western blot. An increase in pTau levels in Tg2576 mice receiving vehicle are shown compared to WT-vehicle mice. Results are expressed as mean ± SEM. (n= 8-10 in each group) (** $p \leq 0.01$). (**b**). Representative Golgi staining images of the apical dendrites on CA1 hippocampal pyramidal neurons in WT and Tg2576 animals treated with vehicle. Scale bar: 10 µm. WT vehicle group showed a significant higher level in the spine density of apical dendrites on hippocampal CA1 pyramidal neurons than Tg2576 vehicle mice (n = 34-36 neurons; *** $p \leq 0.001$).
